# Metastatic MiT family/TFE translocation renal cell carcinoma in adults: case series reports and literature reviews

**DOI:** 10.3389/fonc.2025.1501820

**Published:** 2025-03-19

**Authors:** Xueru Sun, Hong Wang, XiuYue Man, Chen Chen, XiaoFeng Cong, Jing Zhang, Lei Yang

**Affiliations:** Department of Cancer Center, The First Hospital of Jilin University, Changchun, Jilin, China

**Keywords:** Mit family translocation renal cell carcinoma (MiT/TFE tRCC), molecular characterization, genetic mutation, biological behavior, targeted therapy, clinical management

## Abstract

This article presents a case study of three patients diagnosed with MiT/TFE tRCC at our hospital. The tumors were located in the left kidney of all three patients, with two of them being under 30 years old. Within a short timeframe, two of all patients developed liver metastases. Genetic testing was conducted in one case, FISH testing in another, and all cases underwent a combination of targeted therapy and immunotherapy. By analyzing the clinical, pathological, and genomic characteristics of these patients, this article aims to enhance the understanding of MiT family translocation renal cell carcinoma, as well as improve the diagnosis, treatment, and prognosis of this rare form of renal cell carcinoma. Further evidence is provided to support these findings.

## 1 Introduction

MiT family translocation renal cell carcinoma (MiT/TFE tRCC) is a type of renal cell carcinoma that is sporadic and characterized by fusion genes involving the MiT/TFE family. It is classified as MiT/TFE tRCC in the 2016 WHO classification (1, 2). MiT/TFE tRCC is found in 20%-75% of renal cell carcinomas in children and 1%-4% in adults (3). Recent studies suggest that MiT/TFE tRCC may be more aggressive in adults compared to pediatric patients, who typically have a less severe course (4). The clinical behavior of MiT/TFE tRCC in adults varies, with some patients experiencing a slow progression of the disease while others deteriorate rapidly (5). MiT/TFE tRCC presents with varied morphologies, posing challenges in differentiation from clear cell carcinoma. Immunohistochemistry is typically utilized for confirmation of diagnosis, while FISH diagnosis remains the current gold standard (6). Treatment guidelines for metastatic MiT/TFE tRCC lack consensus, with limited data on antitumor efficacy primarily stemming from retrospective studies (7). Due to its rarity, further investigation is warranted to elucidate the biological behavior, pathological characteristics, and their implications on clinical outcomes and prognosis of MiT/TFE tRCC tumors. This article presents a case study of 3 patients diagnosed with metastatic MiT/TFE tRCC and treated at our hospital. Through a detailed analysis of their clinical features, diagnostic approaches, and available treatment modalities, we aim to expand the current understanding of this rare disease and propose novel treatment strategies.

## 2 Case presentation

**Figure 1:**
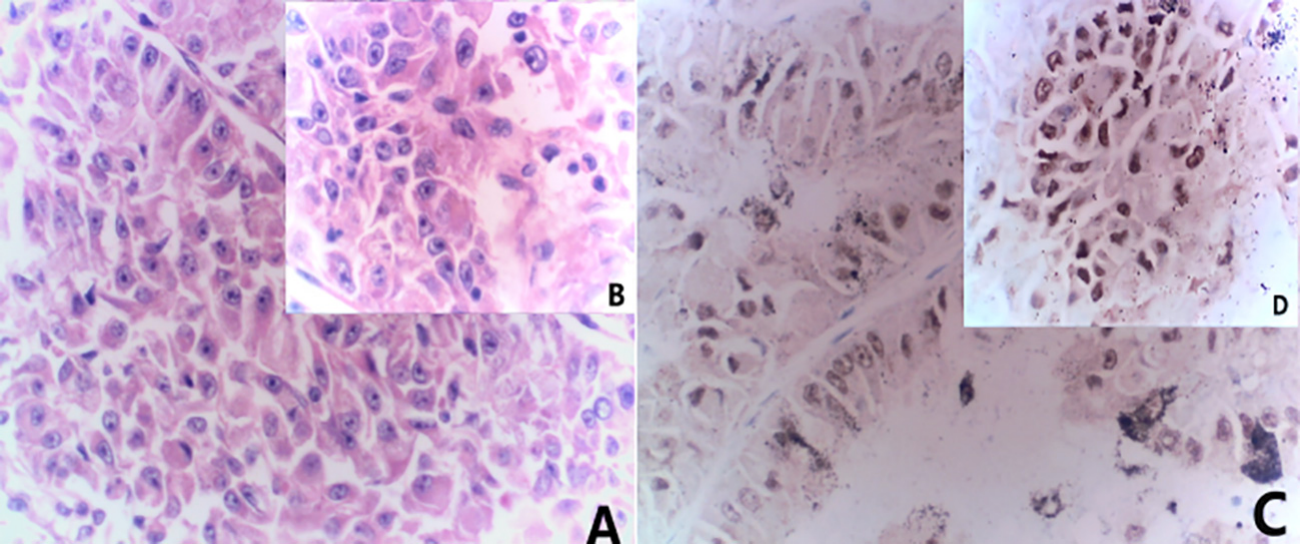
Combined morphological and immunohistochemical results are consistent with Mit family (TFE3/Xp11) translocation renal cell carcinoma. **(A)** HE staining(x20) **(B)** HE staining(x40); **(C)** immunohistochemistry results(x20) showed TFE (+); **(D)** immunohistochemistry results(x40) showed TFE (+).

**Figure 2:**
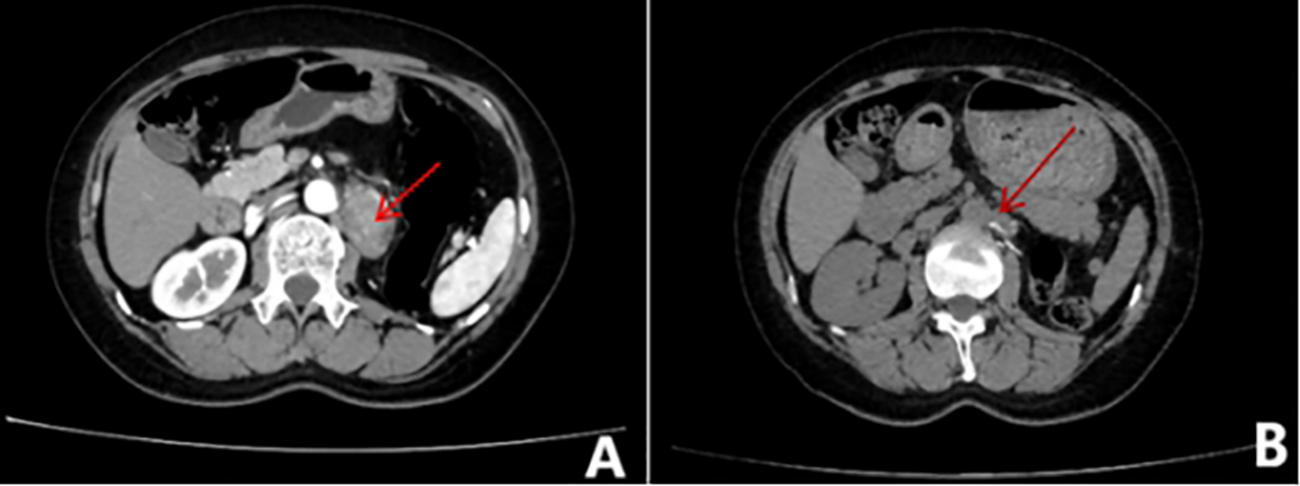
Whole abdominal contrast-enhanced CT scan. **(A)** Before treatment, metastases were visible in the medial left adrenal gland, left paraabdominal aorta, left peritoneum, and omental area. **(B)** After treatment, Metastases can be seen in the parietal peritoneum, omentum, and retroperitoneal space of the left mid-abdominal cavity and on the left side of the abdominal aorta.

The 51-year-old female patient presented at the hospital with complaints of left low back pain and frequent urination persisting for two weeks. Imaging studies revealed a space-occupying lesion in the left kidney, leading to the decision for open left radical nephrectomy and lymphadenectomy. Postoperative pathology confirmed the presence of Mit family (TFE3/Xp11) translocation renal cell carcinoma, immunohistochemistry is shown in (**Figure 1**). The patient underwent regular post-surgery reviews, with CT scans showing multiple intra-abdominal lesions indicating disease progression (**Figure 2A**). Initially, the patient was treated with Topalimab at a dosage of 240 mg as monotherapy; however, this was assessed as progressive disease (PD) after one treatment course(**Figure 2B**). Subsequently, the patient was administered a combination therapy of Axitinib(5 mg twice daily) and Topalimab (240 mg). After four courses of this combined treatment, no new metastatic lesions were identified. The patient treatment timeline can be referenced in **Supplementary Figure 1**.

**Figure 3:**
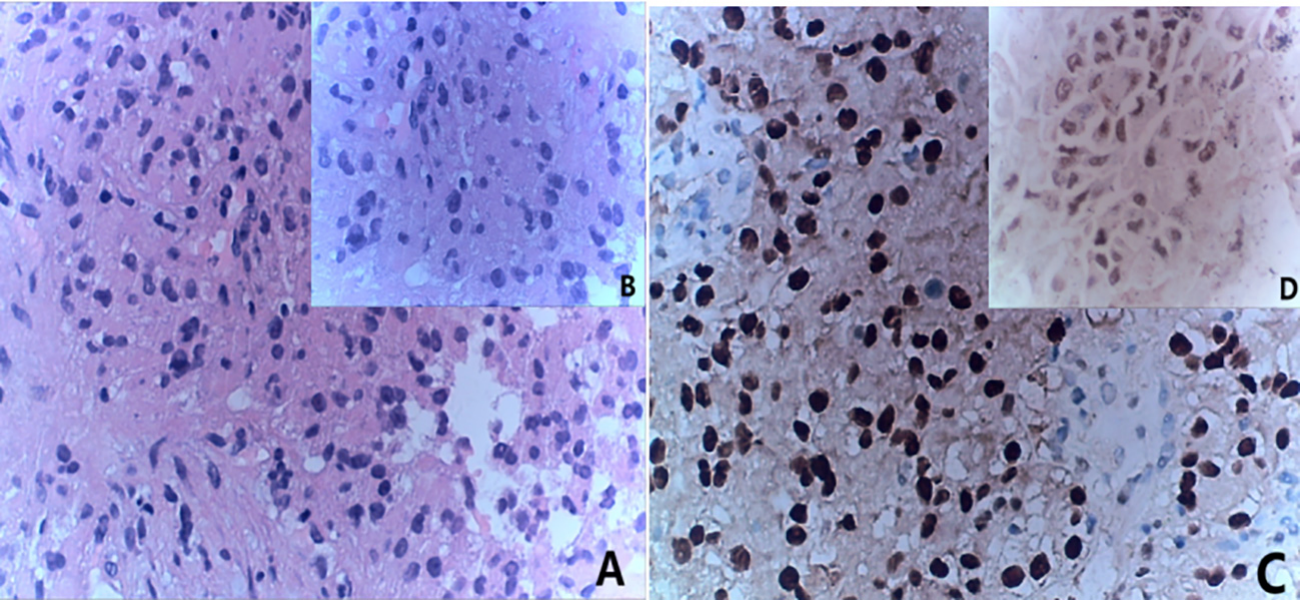
Combined with the results of immunohistochemical staining, it was considered to be metastatic TFE3-rearranged renal cell carcinoma. **(A)** HE staining(x20); **(B)** HE staining(x40); **(C)** immunohistochemistry results(x20) showed TFE (+); **(D)** immunohistochemistry results(x40) showed TFE (+).

**Figure 4:**
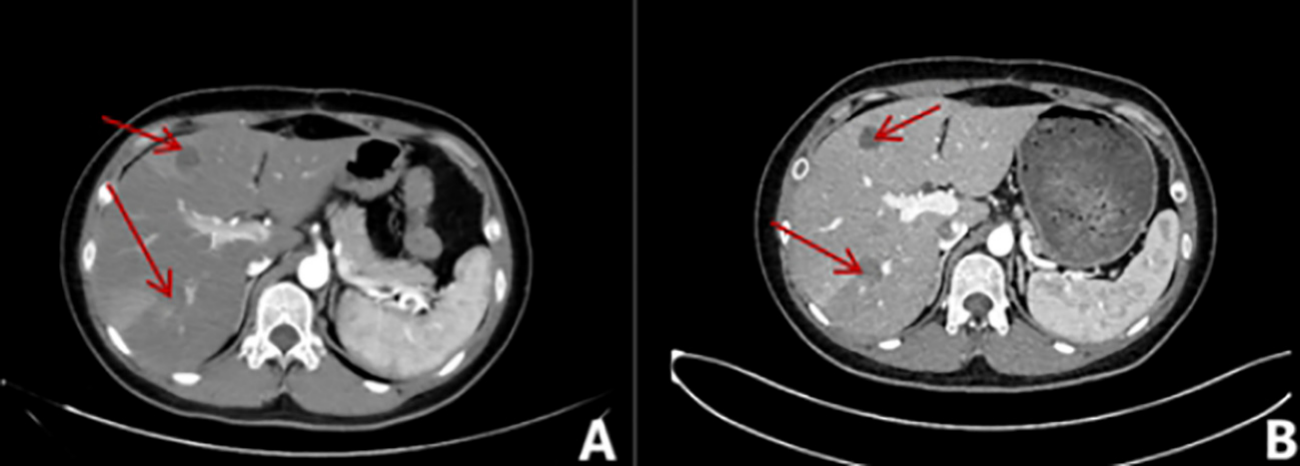
Whole abdominal contrast-enhanced CT scan. **(A)** A round, slightly low-density shadow is present in the right posterior lobe of the liver with unclear borders, measuring approximately 2.2x2.0cm. The edge of the shadow appears to be enhanced in the arterial phase. **(B)** In the right posterior lobe of the liver (IM109), a round-like slightly low-density shadow with unclear boundaries, measuring approximately 2.0cm×1.4cm, was observed. Additionally, there are multiple round-like cystic low-density shadows in the liver, ranging from 0.6cm to 2.4cm in size.

The 28-year-old female patient was admitted to the hospital with complaints of low back pain and hematuria for one day. An abdominal CT revealed a space-occupying lesion in the kidney with rupture and bleeding, leading to laparoscopic left radical nephrectomy. Postoperative pathology confirmed the presence of Mit family (TFE3/Xp11) translocation renal cell carcinoma, immunohistochemistry is shown in (**Figure 3**). The patient’s routine abdominal MRI revealed a single nodule measuring 1.9x1.7cm in the posterior segment of the right lobe of the liver. Subsequent biopsy pathology and immunohistochemistry confirmed the presence of metastatic TFE3 translocation renal cell carcinoma. Two months later, the patient presented with multiple liver metastases (**Figure 4A**). The researchers subsequently utilized conducted high-throughput sequencing of tumor deoxyribonucleotides using paraffin-embedded specimens from the patient’s primary left renal tumor. The results indicated a reduction in CDKN2A copy number and an elevation in MYCN copy number. The patient underwent four cycles of combined therapy, receiving acitinib at a dosage of 5 mg and topalimumab at 240 mg. Following treatment, the patient was assessed according to the Response Evaluation Criteria in Solid Tumors (RECIST) version 1.1. The efficacy evaluation indicated stable disease (SD). (**Figure 4B**). The patient continues to receive ongoing treatment. The patient treatment timeline can be referenced in **Supplementary Figure 1**.

**Figure 5:**
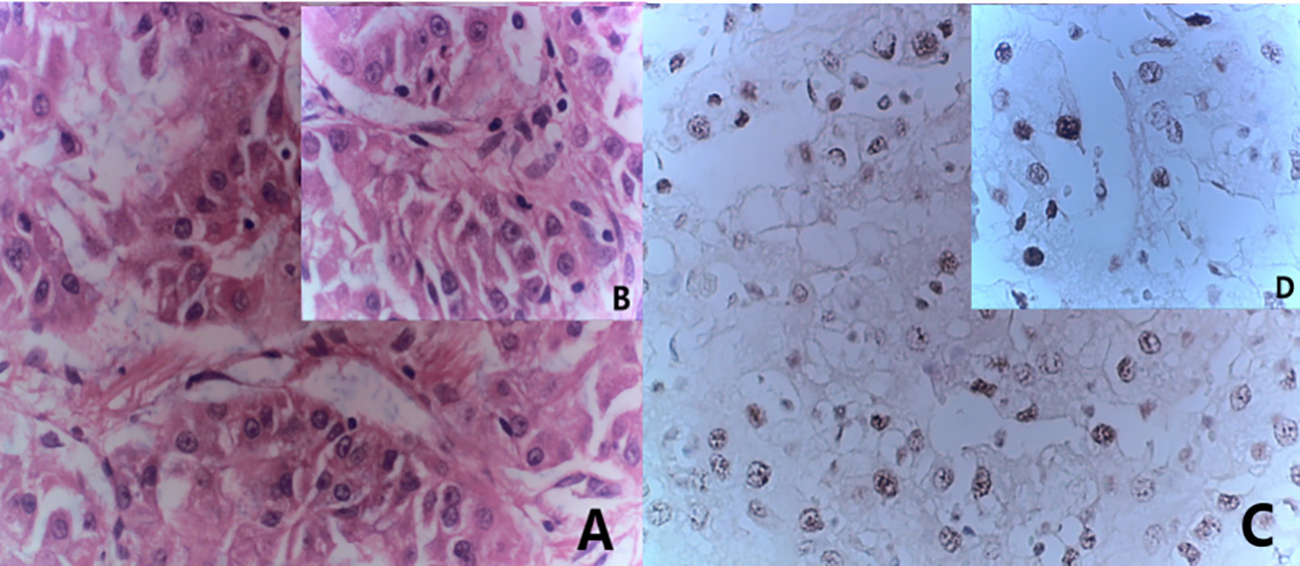
Immunohistochemistry results consistent with MiT family/Xp11 translocation renal cell carcinoma. **(A)** HE staining(x20); **(B)** HE staining(x40); **(C)** immunohistochemistry results(x20) showed TFE (+); **(D)** immunohistochemistry results(x40) showed TFE (+).

**Figure 6:**
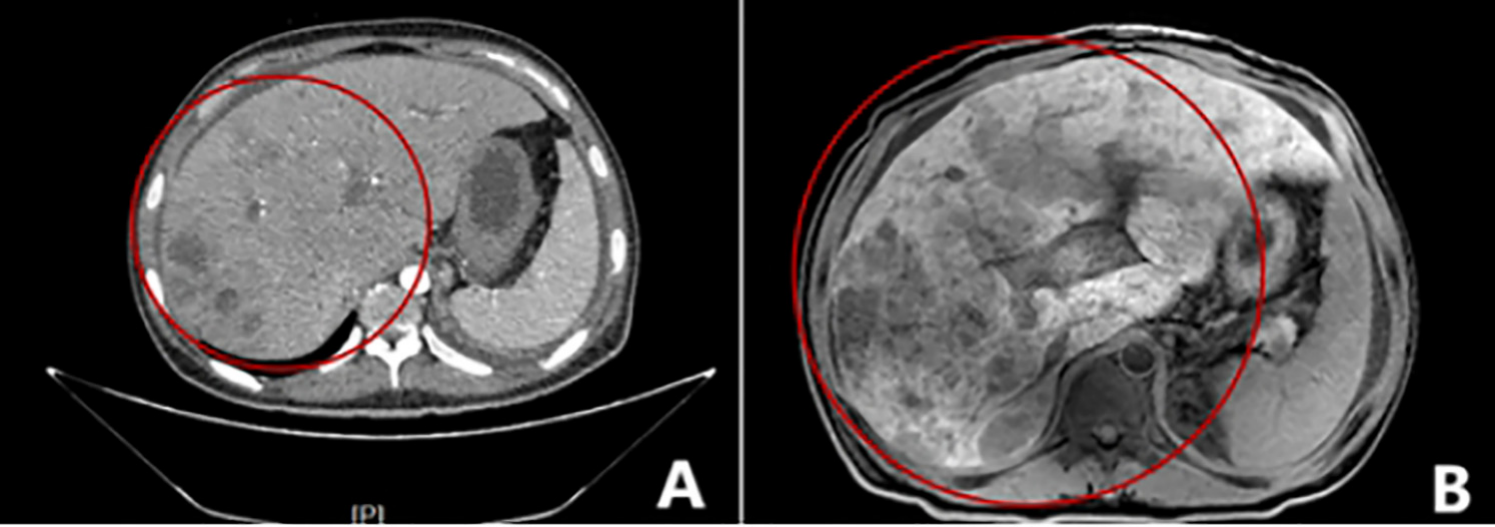
Imaging examination of liver metastases. **(A)** Multiple patchy slightly low-density shadows were seen in the liver parenchyma, and edge intensity enhancement was seen on contrast-enhanced scans; **(B)** Multiple round-like abnormal signal shadows were seen in the liver, approximately 0.3-9.3cm in size, and the edges of the lesions were slightly enhanced on enhanced scans.

The 29-year-old male patient presented to the hospital with complaints of left waist pain and hematuria persisting for one week. An MRI revealed a space-occupying lesion in the middle part of the left kidney, leading to the performance of laparoscopic left radical nephrectomy. Postoperative pathology confirmed the presence of Mit family (TFE3/Xp11) translocation renal cell carcinoma, immunohistochemistry is shown in (**Figure 5**), confirmed by parallel FISH assay showing TFE3-related translocation. Subsequent abdominal MRI revealed multiple space-occupying lesions in the liver, as well as in the left and right branches of the portal vein, along with the presence of tumor thrombi in the main trunk (**Figure 6A**). Following the administration of 4 courses of treatment with [Axitinib 5 mg + Toripalimab 240 mg], there was a further increase in the liver lesions (**Figure 6B**), leading the patient to ultimately discontinue treatment. The patient treatment timeline can be referenced in **Supplementary Figure 1**.

## 3 Discussion

### 3.1 Pathological features

In a comprehensive TCGA case series examining renal cell carcinoma, researchers identified the TFE3/Xp11 translocation in 1.2% of RCC cases (8). Describing the specific clinical manifestations of MiT/TFE tRCC can be challenging, with the key diagnostic criteria often stemming from its distinctive pathological features. The three patients examined in this study all presented with MiT/TFE tRCC displaying a distinct histological appearance, featuring polygonal-shaped tumor cells with hyaline eosinophilic cytoplasm. Immunohistochemical analysis (**Table 1**) revealed that all cases were positive for PAX8 and CK pan, while most were negative for vimentin and cytokeratin 7 (CK7). Additionally, CD10 and methylacyl-CoA racemase (P504S) were both expressed. Case 2 patients exhibited a partial immune response to the melanocyte marker HMB45. Previous studies have suggested that this expression may be attributed to the role of MitF as a melanocyte lineage-specific transcription factor (9). Notably, immunohistochemistry results for this young female patient indicated the presence of both Ksp-cadherin and CD34. While literature reports suggest that MiT/TFE tRCC rarely displays Ksp-cadherin, a marker commonly used in diagnosing chromophobe RCC, the co-expression of these markers in this case may imply a more aggressive biological behavior akin to chromophobe RCC (10). Studies have found that Xp11.2 translocation RCC is a highly vascularized solid RCC characterized by extensive microangiogenesis and lymphangiogenesis. CD34 is identified as the most appropriate vascular marker for microvessel count in Xp11.2 translocation RCC and is linked to prognosis (11). Immunohistochemistry can distinguish MiT/TFE tRCC from other types of renal cancer. While most clear cell RCCs typically show diffuse expression of epithelial markers (cytokeratin and EMA), MiT/TFE tRCC usually displays negative or weakly positive results. The relatively low expression of cytokeratin and vimentin aids in distinguishing MiT/TFE tRCC from tRCC (12). Research has demonstrated that the TFE3 FISH assay serves as a valuable supplementary tool in confirming the diagnosis of MiT/TFE tRCC (13). It is particularly beneficial for cases of MiT/TFE tRCC that present with negative or equivocal pathological or clinical characteristics, as these can be further evaluated through the TFE3 FISH assay (14). The patient in Case 3 underwent FISH testing using TFE3 dual-color probe counting, which detected red and green separation signals. A total of 100 tumor cells were detected, clearly showing TFE3-related translocation. Unfortunately, due to the poor preservation of archived tissue, Cases 1 and 2 could not undergo FISH testing for verification. However, all cases exhibited typical morphological characteristics of TFE3 tRCC and diffuse nuclear positivity for TFE3, consistent with WHO classification diagnostic criteria. The lack of further FISH validation in these cases represents a limitation. The cases we have reported also highlight the importance of conducting prospective FISH validation in clinical practice when immunohistochemistry suggests Mit family translocation renal cell carcinoma, in order to establish precise molecular-level correlations.

**Table 1:** Immunohistochemical characteristics.

	Case 1	Case 2	Case 3
CK pan	focal+	+	focal+
TFE3	+	+	+
Vimentin	–	+	part+
CD10	+	+	+
P504S	+	+	+
PAX8	+	+	+
CA IX	–	focal+	part+
SDHB	+	+	+
HMB45	–	part+	–
CK7	–	focal+	–
CD34	–	+	–

### 3.2 Biological behavior

Clinical behavior in adults with MiT/TFE tRCC can vary significantly, with studies indicating that advanced TNM stage due to pT status is closely linked to poorer progression-free survival (PFS) and overall survival (OS) (15). The article discusses three patients, two of whom are young with disease-free survival (DFS) of 1 month and 10 months, respectively, all of whom developed liver metastases. It is evident from the patient characteristics (**Table 2**) that the TNM stage of these two patients is more advanced compared to the first patient, indicating a more aggressive clinical behavior. Notably, one male patient had the highest postoperative pathological stage among the three, potentially contributing to the rapid development of multiple liver metastases. Genomic changes play a significant role in the increased invasiveness of translocation renal cell carcinoma. Sun et al. conducted gene enrichment analysis, revealing potential epigenetic dysregulation in this disease (16). The genetic testing of the patient in case 2 showed a reduction in the copy number of CDKN2A and an increase in the copy number of the MYCN gene. In previous studies involving gene-level copy number analysis, the sole recurrent focal alteration identified in translocation renal cell carcinoma was the homozygous deletion of the CDKN2A/2B locus (9p21.3), occurring in 19.2% of cases (17). Specifically, a clinical study highlighted that reduced somatic copy number of the CDKN2A gene was linked to larger tumors, higher tumor stage, presence of tumor necrosis and microvascular invasion, and could independently predict prognosis (18, 19). The MYCN gene is a disease-causing gene that promotes cell proliferation and apoptosis (20). MYCN gene mutations are predominantly found in neurogenic cancers and are infrequently observed in renal cell carcinoma. Research has indicated that the silencing of MYCN can promote cell migration, while the presence of MYCN and CDKN2A correlates with tumor stage, metastasis, and overall survival in renal cell carcinoma (21). Furthermore, investigations suggest that copy number variations may precede somatic mutations in TFE3-tRCC, potentially influencing the aggressive nature and prognosis of this subtype (22). The disease-free survival (DFS) of case 2 in this study was only 1 month, and liver lesions progressed again 2 months later. We attribute this to their unique genetic mutation. However, due to the limited number of cases in this study, a large-scale comprehensive genomic analysis could not be conducted. The relationship between somatic copy number alterations (SCNA) and survival outcomes requires further investigation to better understand the significant heterogeneity of TFE3-tRCC.

**Table 2:** Patient characteristics.

	Case 1	Case 2	Case 3
Age	51	28	29
Primary tumor	Left kidney	Left kidney	Left kidney
Tumor volume	6.5cm*6cm*6cm	18cm*16cm*9cm	5cm*4.5cm*4cm
Pathological staging	T1bN1	pT2b	T3aN1
Metastasis sites.	Multiple abdominal metastases	Solitary liver metastasis	Multiple metastases to liver and portal vein.
DFS	18 months	1 month	10 months
FISH	–	–	Unambiguous detection of TFE3-related translocations
Treatment	Axitinib+Toripalimab	Axitinib+Toripalimab	Axitinib+Toripalimab

### 3.3 Treatment and prognosis

Appropriate treatment strategies for MiT/TFE tRCC are currently unclear. Surgery is the preferred treatment for patients with localized tumors, including those with positive regional lymph nodes (3). The three patients discussed in this article were all in the localized stage at diagnosis and underwent radical nephrectomy. For patients with distant metastases, some retrospective studies and single case reports have shown responses to treatment with vascular endothelial growth factor receptor-tyrosine kinase inhibitors (VEGFR-TKIs) and mTOR inhibitors. However, outcomes from different series vary, with studies suggesting that multimodal treatment may help extend survival in patients with advanced disease (23, 24). The introduction of combination therapies using immune checkpoint inhibitors, along with a deeper understanding of the molecular characteristics of RCC, has sparked interest in utilizing immunotherapy for this rare disease. A study revealed promising results with the ICIs/VEGFR-TKI combination in MiT/TFE tRCC patients (25). In this article, all three patients received a combination of Axitinib and Toripalimab, leading to disease stabilization in 2 patients after 4 treatment courses. Previous research has also highlighted the advantages of Axitinib, a second-generation VEGF-targeting drug. The median PFS for initial treatment was 9.4 months, while subsequent first-line treatment yielded a median PFS of 7.4 months. The group receiving ICI/VEGFR-TKI demonstrated significantly longer median PFS and median OS compared to other VEGFR-TKI groups (25). These findings suggest that combining targeted therapy with immunotherapy could become a standard approach for treating metastatic MiT/TFE tRCC in the future.

## 4 Conclusion

This article presents 3 cases of MiT/TFE tRCC, outlining the immunohistochemical features that aid in clinical diagnosis and differentiation. The aggressive nature and prognosis of MiT/TFE tRCC are closely intertwined. Furthermore, the positive outcomes observed with targeted therapy and immunotherapy in 2 cases offer valuable insights for the clinical management of metastatic MiT/TFE tRCC.

## Data Availability

The original contributions presented in the study are included in the article/**Supplementary Material**. Further inquiries can be directed to the corresponding author.

## Supplementary material

Supplementary Figure 1 Patient Treatment Timeline.
