# Supplementary figures and images for: Metastatic MiT family/TFE translocation renal cell carcinoma in adults: case series reports and literature reviews
Source: Front Oncol. 2025 Mar 19;15:1501820. doi: 10.3389/fonc.2025.1501820 (PMC11961414; doi:10.3389/fonc.2025.1501820)

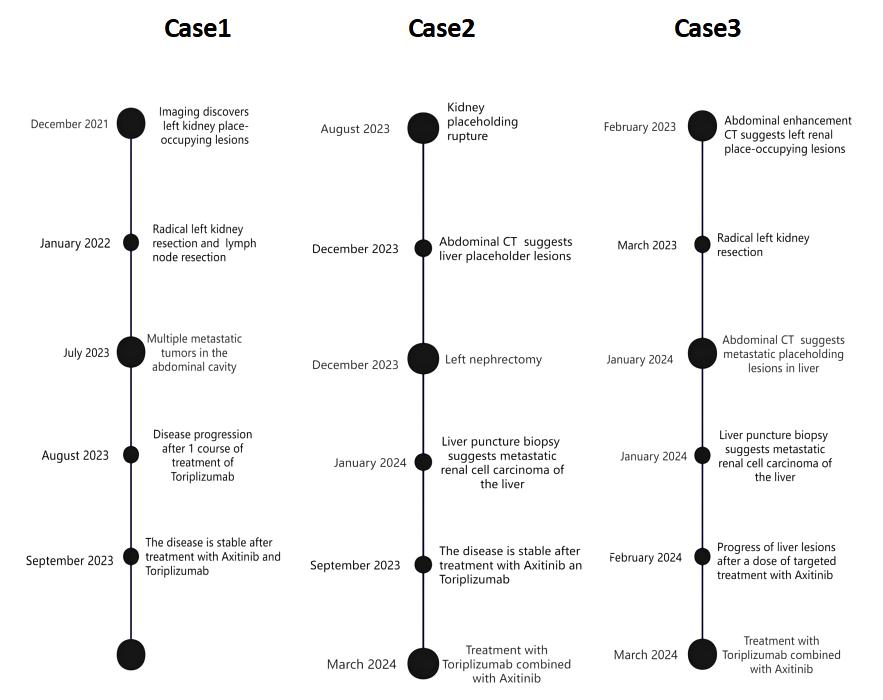

Supplement: Supplementary Figure 1 — Patient Treatment Timeline. [file Image1.jpeg]
